# Mutualistic Coupling Between Vocabulary and Reasoning Supports Cognitive Development During Late Adolescence and Early Adulthood

**DOI:** 10.1177/0956797617710785

**Published:** 2017-08-08

**Authors:** Rogier A. Kievit, Ulman Lindenberger, Ian M. Goodyer, Peter B. Jones, Peter Fonagy, Edward T. Bullmore, the Neuroscience in Psychiatry Network, Raymond J. Dolan

**Affiliations:** 1 Max Planck UCL Centre for Computational Psychiatry and Ageing Research, London, England, and Berlin, Germany; 2 MRC Cognition and Brain Sciences Unit, University of Cambridge; 3 Max Planck Institute for Human Development, Berlin, Germany; 4 Department of Psychiatry, University of Cambridge; 5 Cambridgeshire and Peterborough National Health Service Foundation Trust, Cambridge, United Kingdom; 6 Research Department of Clinical, Educational and Health Psychology, University College London; 7 ImmunoPsychiatry, GlaxoSmithKline, Stevenage, United Kingdom; 8 Medical Research Council/Wellcome Trust Behavioural and Clinical Neuroscience Institute, University of Cambridge; 9 The Wellcome Centre for Human Neuroimaging, University College London

**Keywords:** cognitive development, mutualism, vocabulary, fluid reasoning, longitudinal modeling, open data

## Abstract

One of the most replicable findings in psychology is the *positive manifold*: the observation that individual differences in cognitive abilities are universally positively correlated. Investigating the developmental origin of the positive manifold is crucial to understanding it. In a large longitudinal cohort of adolescents and young adults (*N* = 785; *n* = 566 across two waves, mean interval between waves = 1.48 years; age range = 14–25 years), we examined developmental changes in two core cognitive domains, fluid reasoning and vocabulary. We used bivariate latent change score models to compare three leading accounts of cognitive development: *g*-factor theory, investment theory, and mutualism. We showed that a mutualism model, which proposes that basic cognitive abilities directly and positively interact during development, provides the best account of developmental changes. We found that individuals with higher scores in vocabulary showed greater gains in matrix reasoning and vice versa. These dynamic coupling pathways are not predicted by other accounts and provide a novel mechanistic window into cognitive development.

Among the most reproducible findings in the literature on general cognitive ability is the *positive manifold*—the pervasive positive correlation between distinct cognitive abilities (Deary, 2012; Spearman, 1927). The positive manifold allows the extraction of a single factor, general intelligence (*g*), which summarizes a considerable proportion of shared variance across abilities within a single index; *g* has remarkable predictive ability for a variety of life outcomes, including health, income, mortality, mental health, educational attainment, and socioeconomic status (Deary, 2012). Although the existence of a positive manifold and the *g* factor as a statistical entity is beyond question, its ontology and ontogeny are more contentious.

One challenge arises out of the fact that the *g* factor is almost always derived from cross-sectional data, and this can obscure developmental patterns that are not adequately accounted for in many influential theories. For instance, van der Maas et al. (2006) has noted that one of the most influential modern works on the *g* factor (Jensen, 1998) fails to address the issue of development, despite observations of a relatively rapid increase in higher cognitive abilities such as reasoning, knowledge, and mental speed during childhood and adolescence, a trajectory mirrored by an increasingly steep decline in these abilities during old age (Deary, 2012). Moreover, very different hypotheses regarding the underlying nature of *g* can give rise to mathematically equivalent statistical patterns in cross-sectional data (van der Maas et al., 2006).

Here, we asked whether a lack of attention to development has limited a comprehensive understanding both of the *g* factor and its development over time. Life-span changes in cognitive abilities provide a crucial inroad into the ontological status of *g*, which enables one to ask whether there truly is an underlying general factor that plays a causal role during cognitive development or, alternatively, whether a positive manifold arises out of a more complex developmental process. We considered three possible accounts of cognitive development: *g*-factor theory, investment theory, and mutualism, each of which provides a distinct causal account of the emergence of cognitive abilities during development. Crucially, developments in structural equation modeling (McArdle & Hamagami, 2001) allow each of these accounts to be translated into psychometric models, which enabled us to compare them directly using the same longitudinal data set.

The first account, *g*-factor theory (Gignac, 2014; Jensen, 1998), posits that a single underlying general ability is used in various domains. For example, Gottfredson (2002) states that “*g* is a highly general capability for processing complex information of any type” (p. 25). A simple developmental perspective based on the *g* factor proposes that during early development, an individual’s general ability increases over time, which in turn yields increased scores across a variety of abilities that depend directly or indirectly on *g*. A defining feature of this account is an absence of direct causal links between cognitive abilities. Evidence for this *g*-factor account comes from Gignac (2014, 2016), who suggested that the *g*-factor structure is relatively stable between the ages of 2.5 and 10 years (Gignac, 2014) and that the residual structure of lower cognitive factors is more compatible with *g*-factor theory than with competing accounts, such as mutualism (Gignac, 2016). Contrary evidence comes from McArdle, Ferrer-Caja, Hamagami, and Woodcock (2002), who showed that developmental trajectories across abilities vary considerably not just in their developmental order but also in their shape; they conclude that “a single *g* factor yields an overly simplistic view of growth and change over age” (p. 115).

A second influential account is Cattell’s investment theory (Cattell, 1971). This is based on a division of cognitive abilities into crystallized abilities (knowledge-based) and fluid abilities (flexible skills not dependent on acquired knowledge or skills). The theory is based on a central developmental claim, namely that fluid abilities are invested in order to acquire crystallized abilities. Recent work (Weiland, Barata, & Yoshikawa, 2014) suggests that executive-function scores at the beginning of a preschool year predict improvements in vocabulary performance at the end of the year but not vice versa. Research on a large cross-sectional sample (Valentin Kvist & Gustafsson, 2008) found that the factor structure of general and fluid abilities within and across groups was compatible with investment theory. However, these findings are ambiguous (Valentin Kvist & Gustafsson, 2008), and other researchers found no such effect (Christensen, Batterham, & Mackinnon, 2013), only the reverse pattern (Fuhs & Day, 2011) or an effect only in one cohort (Ferrer & McArdle, 2004). Similarly, Schmidt and Crano (1974) used cross-lagged panel analysis to test investment theory but found evidence that both crystallized and fluid abilities are related over time, concluding that investment theory cannot account for this pattern.

A third developmental account is the *mutualism* model. This model suggests causal interactions between multiple basic cognitive abilities across developmental time, such that cognitive abilities mutually facilitate longitudinal growth. Under this assumption, developmental change will yield a positive manifold even from a starting point of completely uncorrelated cognitive abilities. The model predicts positive coupling between multiple basic cognitive abilities across early development. The strongest empirical evidence for mutualistic processes comes from a life-span cohort study that observed longitudinal coupling effects among multiple cognitive domains, including those associated with speed, memory, and vocabulary (McArdle, Hamagami, Meredith, & Bradway, 2000, pp. 67–68). Contrary evidence from a cross-sectional sample suggests that an increase in *g*-factor strength expected in the strongest version of mutualism is not unambiguously observed (Gignac, 2014).

Several challenges preclude strong inferences regarding the best model of cognitive development. First, the studies discussed in the preceding paragraphs drew their samples from various points in the life span, which may be governed by different developmental mechanisms. Second, several reports have relied on statistical techniques such as cross-lagged panel models (Schmidt & Crano, 1974) not ideally suited to study change. Third, other studies have relied on cross-sectional cohorts, which limits the range of inferences that can be made (e.g., Gignac, 2014; Valentin Kvist & Gustafsson, 2008). Most important, although several studies tested specific theories (e.g., Ferrer & McArdle, 2004; Ghisletta & Lindenberger, 2003; McArdle et al., 2002; McArdle et al., 2000), to the best of our knowledge, no study has directly compared these three prominent accounts of development. Our aim in this study was to fill this gap by exploiting innovations in structural equation modeling (McArdle & Hamagami, 2001) that are uniquely suited to directly compare these three accounts. To do this, we exploited data from a large developmental cohort measured on two domain-representative (crystallized and fluid) standardized subtests, Matrix Reasoning and Vocabulary from the second edition of the Wechsler Abbreviated Scale of Intelligence (WASI-II; Wechsler, 2011). Using a latent change score (LCS) framework, we modeled the three theoretical accounts of change in cognitive abilities as three different LCS models.

## Method

### Sample

We recruited 785 participants (402 female, 383 male; mean age: 19.05 years, range: 14.10–24.99) for the University of Cambridge-University College London Neuroscience in Psychiatry Network (NSPN) cohort. This sample size has been shown to be sufficient to fit moderately complex structural equation models with adequate power (e.g., Wolf, Harrington, Clark, & Miller, 2013). We tested 566 of these participants a second time, on average 1.48 years later (range: 0.65–2.62 years). Those who returned for a second wave did not differ significantly from those who did not return on Time 1 Vocabulary scores, *t*(366.5) = 0.27, BF01 = 10.86,1 as well as on Time 1 Matrix Reasoning scores, *t*(361.57) = 0.54, BF_01_ = 9.64; sex, χ^2^(1, *N* = 785) = 0.7254, BF_01_ = 8.11, and current or past treatments for emotional, behavioral, or mental health problems—current: *t*(271.6) = −1.47, BF_01_ = 2.08, past: *t*(348.04) = −0.95, BF_01_ = 6.8. These groups also did not significantly differ in terms of parental education—i.e., the age at which their mothers left school, *t*(156.51) = −0.85, BF_01_ = 4.93, or fathers left school, *t*(159.4) = −0.49, BF_01_ = 4.93. Participants with complete data were slightly younger at the time of first testing (*M* = 18.81 years) than those with incomplete data (*M* = 19.67 years), *t*(415.62) = −3.77, BF_10_ = 64.7, and had slightly higher scores on the Barratt Impulsiveness Scale (BIS, Version 11; Stanford et al., 2009; *M*s = 63.30 vs. 60.52, respectively), *t*(389.9) = −3.58, BF_10_ = 46.77. Implementing either complete case analysis or excluding individuals with BIS scores above a cutoff of 74 (see Stanford et al., 2009, p. 387) did not meaningfully affect the model parameters or model comparisons reported here. The role of age is discussed in more detail in the Results. Prior to the study, full ethical approval was obtained from the University of Cambridge Central Ethics Committee (Reference No. 12/EE/0250).

### Measures

Participants were tested using the Matrix Reasoning and Vocabulary subtests from the WASI-II. Matrix Reasoning measures fluid and visual intelligence by means of a series of incomplete visual matrices; participants pick one out of five options that best completes the matrix. The Vocabulary subtest measures participants’ breadth of word knowledge and verbal concepts; examiners present words or concepts orally and ask participants to verbally define and describe them. Both subtests have excellent interrater reliability (*r*s = .98 and .95), split half reliability (*r*s = .90 and .92), and concurrent validity (*r*s = .71 and .92) with comparable tests, such as the fourth editions of the Wechsler Intelligence Scale for Children (WISC-IV) and the Wechsler Adult Intelligence Scale (WAIS-IV; key reliability and validity statistics are summarized in McCrimmon & Smith, 2013, p. 339). The highly similar reliabilities of the measures ensured comparable interpretation of cross-domain effects. Prior to further modeling, scores on both tests at Time 2 were rescaled to control for varying intertest intervals, as proposed by Ferrer and McArdle (2004).

### Modeling framework

To tease apart candidate mechanisms of development, we fitted a series of LCS models (Kievit et al., 2017; McArdle & Hamagami, 2001; McArdle et al., 2000). These models conceptualize differences between successive measurements as latent change factors. Crucially, this allowed us to directly model within-subjects changes as a function of structural parameters, which made these models more suitable for our purposes than latent growth curve models (McArdle & Hamagami, 2001). The basic equation of the LCS model specifies the score of person *i* on test *Y* at time *t* as a sum of the score at time *t* – 1 and a change, or difference, score as follows:

$$Y_{i,t}\;=\;\beta_{t,t-1}Y_{i,t-1}\;+\;\Delta Y_{i,t}.$$

A key step in the LCS model specification is to set the regression weight β_*t, t–1*_ to 1 (McArdle & Hamagami, 2001), which allowed us to rewrite the change scores as follows:

$$\Delta Y_{i,t}=Y_{i,t}-Y_{i,t-1}.$$

These change scores were then modeled as perfect indicators of a latent factor of change scores. In cases in which there was only one observed variable, or indicator, per construct, the LCS factor was construed as the difference between these indicators over time. In the absence of coupling, the intercept of the simple LCS model gives approximately identical results as a paired-samples *t* test when comparing differences across two measurement occasions, but it allows two additional parameters of considerable theoretical importance to be modeled: the variance in change scores (i.e., whether individuals change homogeneously or not over time) and the covariance between scores at *t* – 1 and change scores. We extended the basic univariate LCS model to a bivariate LCS model with abilities *Y*1 and *Y*2 (McArdle et al., 2002) by modeling the change scores on two domains *Y*1 and *Y*2 (Vocabulary and Matrix Reasoning scores) as the function of two processes: a self-feedback process (β) and a coupling process (γ), as follows:

$$\Delta Y1_{i,t}\;=\;\;\beta 1Y1_{i,t-1}\;\;+\;\;\gamma 1Y2_{i,t-1}\;.$$

The self-feedback parameter (β) is thought to reflect a combination of effects, including regression to the mean and a dampening effect induced by an end horizon for rapid development (i.e., individuals reaching their performance ceiling). The coupling parameter (γ) is of special importance for several developmental accounts. It captures whether the change in *Y*1 is determined by the *t* – 1 scores in *Y*2 (and vice versa) and thus indexes the degree to which change in one domain is affected by the level of a cognitive ability in some other domain, above and beyond the self-feedback parameter. These gammas are conceptually similar to the M matrix in the mutualism model, which captures the strengths of the interactions between abilities in a complex system (van der Maas et al., 2006, p. 845).

**Fig. 1.**
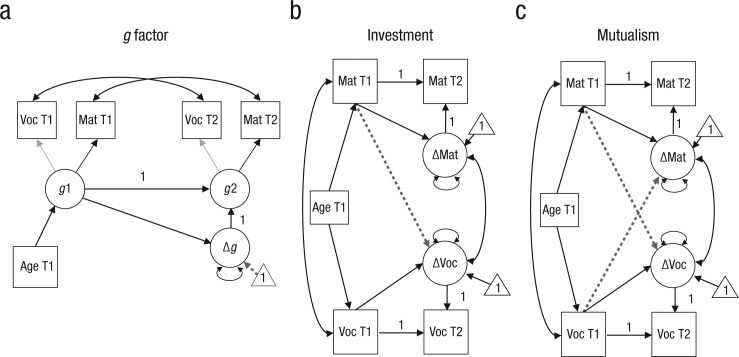
Illustrations of the (a) *g*-factor, (b) investment, and (c) mutualism models. In each model, age at Time 1 (T1) was entered as a covariate of Vocabulary (Voc; capturing crystallized abilities) and Matrix Reasoning (Mat; capturing fluid abilities) scores at T1. Both tests were taken from the second edition of the Wechsler Abbreviated Scale of Intelligence (Wechsler, 2011). Circles indicate latent variables, and rectangles indicate observed variables. Thick single-headed arrows indicate regressions. Double-headed arrows indicate variance and covariance. Key parameters are indicated by dashed arrows, and triangles denote intercepts. A “1” indicates which values were constrained to unity. Factor loadings in the *g* model were equality-constrained across measurement occasions (thin single-headed arrows). T2 = Time 2.

We fitted models for *g*-factor theory, investment theory, and mutualism. First, for the *g*-factor model (Fig. 1a), we conceptualized observed scores on Vocabulary and Matrix Reasoning as a function of an underlying *g* score for each time point.

Second, investment theory implies that scores in fluid abilities (here indexed by Matrix Reasoning scores) should positively influence the degree of change in crystallized abilities (indexed by Vocabulary scores), such that individuals with greater fluid ability will, on average, improve more in crystallized abilities than peers with lower Matrix Reasoning scores at Time 1. This process was modeled by a single coupling parameter from Matrix Reasoning scores at Time 1 on the Vocabulary change factor at Time 2 (Fig. 1b). Finally, the mutualism model (Fig. 1c) predicts bivariate coupling between both cognitive abilities; specifically, higher starting points in vocabulary would lead to larger gains in matrix reasoning and vice versa. In all models, we added age as a covariate to account for differences in baseline scores but did not include age anywhere else in the model (i.e., we hypothesized that the dynamics of change were fully captured by the change dynamics proposed by each theory).

### Model fit and comparison

Models were estimated in the lavaan software package (Version 5.22; Rosseel, 2012) using full information maximum likelihood with robust standard errors to account for missingness and nonnormality. No observations were excluded. We assessed overall model fit via the chi-square test, the root-mean-square error of approximation (RMSEA; acceptable fit: < .08, good fit: < .05), the comparative fit index (CFI; acceptable fit: .95–.97, good fit: > .97), and the standardized root-mean-square residual (SRMR; acceptable fit: .05–.10, good fit: < .05; Schermelleh-Engel, Moosbrugger, & Müller, 2003). We compared the three models in three ways: overall model fit (cf. Schermelleh-Engel et al., 2003), information criteria (viz., Akaike’s information criterion, AIC, and Bayesian information criterion, BIC), and Akaike weights (Wagenmakers & Farrell, 2004), which use differences in AICs to quantify the relative likelihood of a model being the best among the set of competitors, given the data.

## Results

**Fig. 2.**
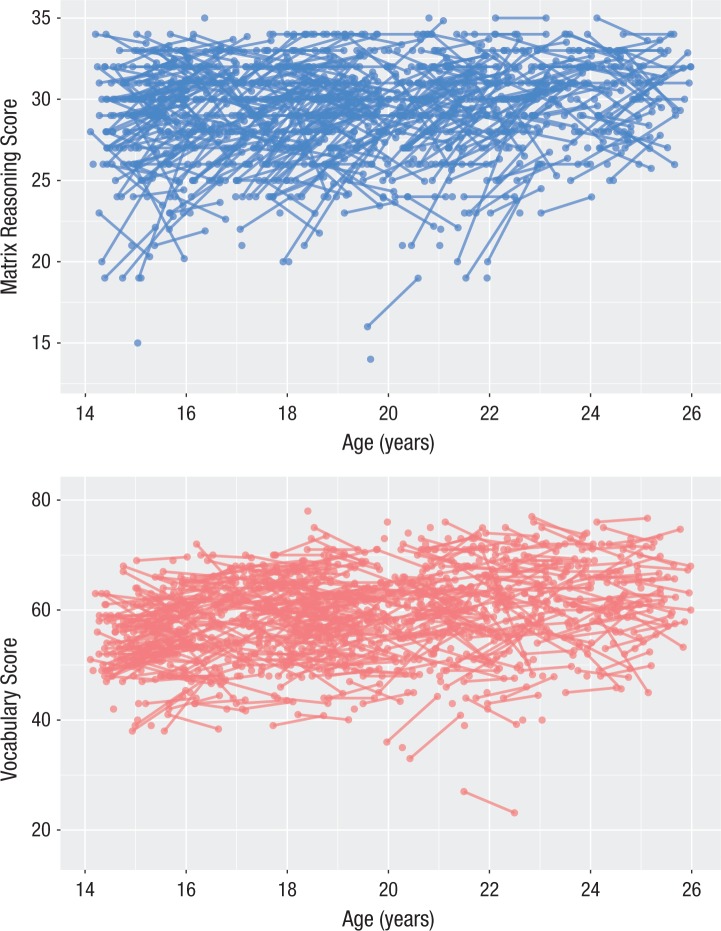
Scatterplots showing the association between age and score on the Matrix Reasoning subtest (top) and Vocabulary subtest (bottom) of the second edition of the Wechsler Abbreviated Scale of Intelligence (Wechsler, 2011). Lines connect the rescaled scores of those individuals who completed the test at both waves.

Raw scores and descriptive statistics for the Matrix Reasoning and Vocabulary subtests are shown in Table 1, and the association between age and score on each test is shown in Figure 2. Before fitting the models shown in Figure 1, we fitted two univariate LCS models to Vocabulary and Matrix Reasoning scores in order to quantify change within each domain. Both models fitted the data well: Matrix Reasoning: χ^2^(1) = 2.59, *p* = .108; RMSEA = .045, 90% confidence interval (CI) = [0.000, 0.114]; CFI = 0.996; SRMR = 0.013; Yuan-Bentler scaling factor = 0.917; Vocabulary: χ^2^(1) = 0.033, *p* = 0.85; RMSEA = 0.00, 90% CI = [0.000, 0.049]; CFI = 1.0; SRMR = 0.001; Yuan-Bentler scaling factor = 1.052. Both models showed evidence for change over time (unstandardized change-score intercepts^2^—Matrix Reasoning: 10.171, *SE* = 0.769, *z* = 13.22; Vocabulary: 9.0, *SE* = 1.22, *z* = 7.36). Further, both models showed evidence for negative feedback: Higher scores at Time 1 were associated with less improvement at Time 2, a pattern compatible with regression to the mean and developmental-ceiling effects (Matrix Reasoning: −0.331, *SE* = 0.026, *z* = −12.82; Vocabulary: −0.147, *SE* = 0.21, *z* = −7.15). Finally, both models revealed significant evidence for individual differences in change scores (variance of Matrix Reasoning change scores = 2.85, *SE* = 0.23, *z* = 12.73; variance of Vocabulary change scores = 11.67, *SE* = 1.11, *z* = 10.47).

**Table 1.** Raw Scores and Descriptive Statistics for Matrix Reasoning and Vocabulary Scores

		Score					
Task	*N*	Mean	Minimum	Maximum	*SD*	Skewness	Excess kurtosis
Matrix Reasoning Time 1	785	29.04	14	35	3.18	−0.87	1.33
Matrix Reasoning Time 2	565	29.63	17	35	2.88	−0.84	0.85
Vocabulary Time 1	785	58.57	27	78	7.85	−0.26	0.05
Vocabulary Time 2	566	58.99	20	77	7.74	−0.56	1.17

Note: The Matrix Reasoning and Vocabulary subtests were taken from the second edition of the Wechsler Abbreviated Scale of Intelligence (Wechsler, 2011).

Having shown, as expected, a growth in scores in both domains, we next fitted all three models (*g* factor, investment, and mutualism) to determine which provided the best account of longitudinal development in these two cognitive domains across the two measurement occasions. To ensure comparability of factor scores across Time 1 and Time 2 for the *g*-factor model, we tested for longitudinal measurement invariance (Widaman, Ferrer, & Conger, 2010). We found that imposing weak invariance across time points (factor loadings) led to negligible decrease in model fit (ΔCFI = 0.004; Cheung & Rensvold, 2002). Imposing strong invariance (equality of both factor loadings and thresholds) also led to acceptable decrease in model fit (ΔCFI = 0.014). This suggests that longitudinal measurement invariance is tenable, and we interpreted changes in factor scores accordingly. Next, we fitted the investment and mutualism models, which differed only in the presence or absence of a Vocabulary-to-Matrix-Reasoning coupling parameter.

**Fig. 3.**
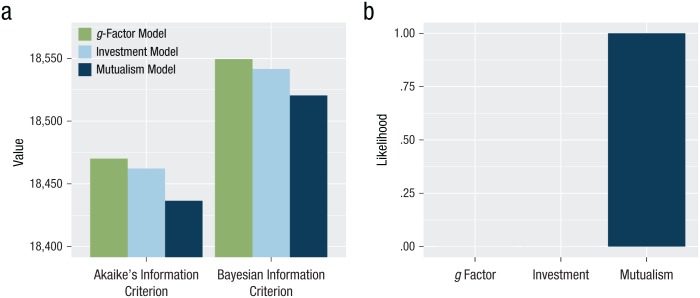
Akaike’s information criterion and Bayesian information criterion (a) and normalized probabilities using Akaike weights (b), for each of the three models.

In Table 2, we report the fit statistics for each of the three competing models. This comparison suggests that the mutualism model fitted the data best, showing excellent model fit on all indices. The two alternative models (investment and *g* factor) showed comparable model fit between each other, and any difference was marginal according to conventional guidelines. As the mutualism model was also the most complex model, we plotted information criteria (AIC and BIC) for each of the three models to explicitly weigh parsimony, as shown in Figure 3a. This comparison showed a superior fit on both indices for the mutualism model. Finally, we computed Akaike weights. These are shown in Figure 3b, which illustrates that the mutualism model has by far the highest normalized probability (> 99.99%) of being the best model given our data. Compared with the other two models, the mutualism model was 1.98 × 10^7^ times more likely to be the best model. As the investment model was nested within the mutualism model, we compared the two with a chi-square test, which again showed that the mutualism model outperformed the investment model, Δχ^2^(1) = 22.75, *p* < .001.

**Table 2.** Fit Statistics for Each of the Three Models

Model	χ2	*df*	RMSEA	CFI	SRMR
*g* factor	30.078	3	0.107 [0.077, 0.140]	0.979	0.029
Investment	26.28	3	0.099 [0.068, 0.135]	0.982	0.039
Mutualism	0.132	2	0.000 [0.000, 0.020]	1.00	0.001

Note: For root-mean-square errors of approximation (RMSEAs), 90% confidence intervals are given in brackets. CFI = comparative fit index; SRMR = standardized root-mean-square residual.

**Fig. 4.**
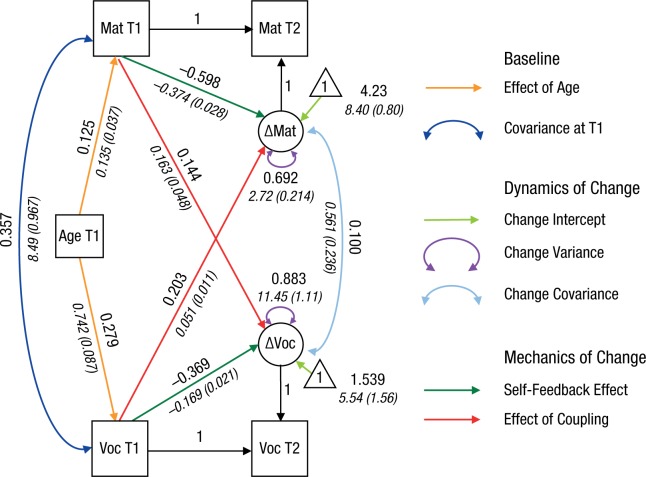
Estimated parameters for the mutualism model. Values in Roman are standardized parameter estimates, and values in italics are unstandardized parameter estimates (with standard errors in parentheses). See Figure 1 for an explanation of the notational system used. Further results are given in Table S1 in the Supplemental Material. Mat = Matrix Reasoning; Voc = Vocabulary; T1 = Time 1; T2 = Time 2.

Having established the superior fit of the mutualism model, we next investigated its estimated parameters in more detail (see Fig. 4; Table S1 in the Supplemental Material available online contains all parameter estimates and 95% confidence intervals). As expected, Matrix Reasoning and Vocabulary scores at Time 1 were positively correlated, and age at first testing predicted scores on both tasks at Time 1. In addition to significant latent change intercepts (i.e., increasing scores), variance of change scores led to a substantial drop in model fit when fixed to 0—Matrix Reasoning: Δχ^2^(1) = 83.16, *p* < .001; Vocabulary: Δχ^2^(1) = 13.44, *p* < .001, which suggests that there were considerable individual differences in change between Time 1 and Time 2. Crucially, as predicted by the mutualism model, both coupling parameters were positive: Individuals who started out with a higher Matrix Reasoning score improved more on Vocabulary and vice versa. The coupling effect from Time 1 Vocabulary scores on gains in Matrix Reasoning scores was of typical size (*r* = .203, *r*^2^ = 4.1%) for individual differences analyses, and the fully standardized estimate of Matrix Reasoning on Vocabulary gains was in the small to typical range (*r* = .144, *r*^2^ = 2.1%; Gignac & Szodorai, 2016). Together, the self-feedback and coupling parameters accounted for 30.8% of the individual differences in Matrix Reasoning score changes and for 11.7% of the individual differences in Vocabulary score changes, which illustrates the considerable importance of longitudinal kinematics in cognitive development. Even in the presence of the bivariate coupling parameters, the residual change scores were still positively correlated. This is compatible with (although not direct evidence for) the idea of additional unmeasured cognitive abilities driving change in both vocabulary and matrix-reasoning ability. Further control analyses suggested that the mutualism model could be equality constrained across sexes without a notable drop in model fit, Δχ^2^(18) = 17.184, *p* = .51.

**Fig. 5.**
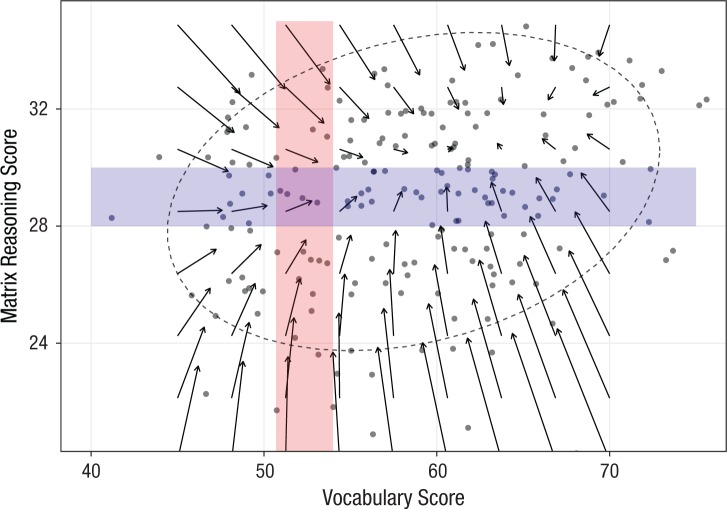
Vector field plot for the mutualism model showing model-implied changes between Time 1 and Time 2. The dots represent the Time 1 Matrix Reasoning and Vocabulary scores of a randomly selected subset of individuals, and each arrow represents a model-implied change between Time 1 (base of arrow) and Time 2 (head of arrow). The horizontal shaded rectangle illustrates the positive effect of higher Vocabulary scores on expected change in Matrix Reasoning scores. The vertical shaded rectangle illustrates that there was a negligible expected Vocabulary improvement for low Matrix Reasoning ability (arrows below 24 on the *y*-axis) but considerable expected vocabulary improvement for individuals with high Matrix Reasoning starting scores (arrows above 28 on the *y*-axis). The dashed ellipse shows the 90% confidence interval for the raw data.

Using Equation 3 and the estimated parameters of the full mutualism model (Fig. 4), we next visualized the expected change between Time 1 and Time 2. To do this, we created a vector field plot (e.g., McArdle et al., 2000, p. 69) in which each arrow represents a (hypothetical) bivariate score at Time 1 (base of each arrow) and model-implied expected score at Time 2 (end of arrow) across a range of possible scores. Figure 5 shows the vector field plot and highlights regions where the mutualistic effects are easiest to see.

Although analytic work (van der Maas et al., 2006) has demonstrated that a *g* factor may arise through mutualism even in the complete absence of individual differences at the beginning of development, we think it most likely that *g*-factor and mutualistic processes operate in tandem. For example, it may be that children show (smaller or larger) consistent individual differences from very early ages (e.g., Gignac, 2014), which are then amplified by developmental processes, such as mutualism. This is in line with previous suggestions of gene-environment interactions whereby initial differences lead to a “reciprocal feedback loop between the phenotype and the environment” that amplifies initial differences (Beam & Turkheimer, 2013, p. 7; see also Briley & Tucker-Drob, 2013; Dickens & Flynn, 2001), a phenomenon observed even in genetically identical mice (Freund et al., 2013). Such models can also reconcile the high heritability of higher cognitive abilities (Briley & Tucker-Drob, 2013) with considerable environmental impacts and may partially reconcile more puzzling facts about heritability and the cultural load of cognitive tasks (Kan, Wicherts, Dolan, & van der Maas, 2013).

In the three models examined here, we included age as a linear covariate to account for individual differences due to age at Time 1 (we will describe alternative parametrizations of age in the Discussion). This reflects a hypothesis that age affects scores at Time 1 but that all aspects of development over time can be captured within the model. Allowing age to directly predict change scores did not improve model fit, Δχ^2^(2) = 0.13, *p* = .93, in line with this hypothesis. Notably, this does not necessarily imply that cognitive development occurs at the same rate across development. The decelerating improvement in late adolescence was captured by the negative self-feedback parameter in Matrix Reasoning and Vocabulary. A second analytic choice is to assume a linear effect of age on scores at Time 1. An age-squared term as predictor of scores at Time 1 could be fixed to 0 without a decrease in model fit, Δχ^2^(2) = 3.79, *p* = .15, which suggests that a linear term would suffice. Third, we included age as a predictor of the raw Vocabulary and Matrix Reasoning scores at Time 1 for the mutualism and investment models but allowed age to predict the *g* factor only in the *g*-factor model (under the assumption that this factor captures the “true” shared variance). Although this is in line with the conceptualization proposed here, we wanted to ensure that this analytic choice did not favor or disfavor the *g* model artificially. We therefore fitted two additional versions of the *g*-factor model by including age either as a covariate of only the observed scores at Time 1 (alternative A) or as covariates of both the observed scores and the *g* factor (alternative B). The mutualism model was preferred to all three conceptualizations of the *g* model—ΔBIC = 28.94 (original *g*-factor model), ΔBIC = 46.17 (alternative A); ΔBIC = 7.09 (alternative B). Together, these analyses suggest that characterizing age as a linear effect was sufficient within this sample, that differences in change scores were not affected by age beyond the indirect effect, and that the mutualism model provided a compelling account of dynamic processes during cognitive development.

## Discussion

In a large (*N* = 785) developmental cohort of adolescents and young adults, we compared three competing accounts that could explain age-related changes in key cognitive abilities. We found that mutualism outperformed alternative accounts based on *g*-factor and investment theory. Specifically, we found evidence for bivariate coupling between Matrix Reasoning scores (as an index of fluid abilities) and Vocabulary scores (as an index of crystallized abilities); specifically, higher starting points in one cognitive domain were associated with greater developmental gains in the other domain. Our findings refine the understanding of cognitive development in several ways. They suggest that covariance between cognitive abilities is, at least in part, a consequence of a developmental process rather than of a single underlying causal entity *g*. Our data provide strong evidence that a model of intellectual development that omits coupling parameters is incomplete.

We can hypothesize several mechanisms to explain the coupling parameters, both direct and indirect. One direct pathway may be that a greater facility with vocabulary and verbal skills allows for swifter, more accurate decomposition of reasoning problems into constituent elements, as well as decreased working memory demands for maintenance of such elements, especially in younger adults. A more indirect pathway, in line with the gene-environment interactions mentioned previously, is that greater vocabulary may be an easily detectable marker of higher cognitive ability, which leads to real-world feedback effects in the form of more academically challenging classes or environments to support perceived ability in a manner that generalizes to other domains. A final, intriguing possibility is that traditionally fluid tasks such as Matrix Reasoning may in fact reflect a hybrid of purely fluid abilities (or learning potential) and more strategic, verbal components akin to crystallized abilities (Kühn & Lindenberger, 2016). This would explain both the life-span trajectories of fluid abilities and the considerable secular gains in fluid abilities in the 20th century (Flynn, 1987).

Our findings suggest a need for a shift away from a narrow focus on desirable cognitive end goals (e.g., adequate performance on abilities such as vocabulary or mathematics) and the incorporation of a simultaneous view across abilities that may have less intrinsic interest but are essential in their capacity to support successful development. For example, skills such as processing speed or working memory may be less important in isolation but may be coupled to other cognitive skills (Kail, 2007), which in turn may affect later life socioeconomic outcomes. In other words, to facilitate early detection and possibly even effective intervention, it may pay off to focus on abilities that have the strongest coupling strengths rather than solely on outcomes that are currently below some desirable threshold. For example, Quinn, Wagner, Petscher, and Lopez (2015) used dynamic models to show that vocabulary was a leading indicator of gains in reading comprehension but not vice versa. Such a finding offers insight into the causal pathways of children with reading difficulties, as well as informing appropriate interventions. Similarly, disruptions to typical development were reported by Ferrer, Shaywitz, Holahan, Marchione, and Shaywitz (2010), who observed that within a subgroup with dyslexia (or “persistently poor readers,” p. 94), the coupling between IQ and reading ability observed in typical groups was absent. This suggests not only a possible mechanism for developmental disorders, but also shows how multivariate longitudinal models can allow for early detection of developmental challenges that are likely to self-reinforce over time.

Although we compared various developmental models and quantified longitudinal coupling, our research has certain limitations. First and foremost, we focused on two cognitive subtests alone, which yielded a relatively simplistic *g* model. Although both are well validated, have highly similar reliabilities, and represent broad cognitive domains, it will be desirable in future studies to represent cognitive abilities by more than one indicator variable and to sample a wider range of cognitive abilities. Our sample was measured on two occasions, and undoubtedly, measurement on more occasions would allow a more precise decomposition of kinetics and kinematics, such as the modeling of lead-lag relations using bivariate dual-change-score models (e.g., Ghisletta & Lindenberger, 2003). Here, we showed that baseline scores are positively associated with cross-domain rates of change. With three or more waves, it is possible to use the change scores at time *t* to predict the change scores at time *t* + 1 (Grimm, An, McArdle, Zonderman, & Resnick, 2012). Moreover, if age is sampled at sufficient frequency, it is possible to examine latent changes as a function of age itself (∆*Y_agei_* ) rather than as testing occasion (∆*Y_ti_* ), which would obviate the need for covariates by binning individuals’ scores by age and estimating models using methods that account for missingness (e.g., Voelkle & Oud, 2017).

An additional challenge with repeated measures data is the improvement in test scores due to practice effects, which may inflate developmental gains or attenuate age-related decline (Rabbitt, Diggle, Smith, Holland, & Mc Innes, 2001; Salthouse & Tucker-Drob, 2008). Although, in our sample, practice effects may have led to greater increases in scores between Time 1 and Time 2, it is unlikely that these effects affected our conclusions regarding mutualism. First, such practice effects would lead to an increase in test scores that are a combination of true (developmental) gains and increases due to practice effects (although see Lövdén, Ghisletta, & Lindenberger, 2004, on the interpretation of practice effects). Notably, if one interprets the gains between Time 1 and Time 2 as a combination of “true” gains and practice effects, this would entail an underestimate of the mutualism effect (as the effect size reflects the prediction of the total gains rather than the non-practice-related gains). In principle, a sufficiently large number of time points spaced at unequal retest intervals would allow for a decomposition of retest effects, but both practical difficulties as well as the inherent collinearity of retest occasions with time intervals has proved methodologically challenging (Hoffman, Hofer, & Sliwinski, 2011).

Finally, we observed our effects in adolescents and young adults, which limited the generalizability of our observations to this developmental period alone. We hypothesize that the coupling effects we observed are likely to be stronger earlier in life and the self-feedback parameters weaker, as developmental change in higher cognitive abilities is most rapid during pre- and early adolescence. Considering these effects at the other end of the life span yields several intriguing questions. It is conceivable that mutualism occurs only during early development, with other processes and mechanisms taking over after initial peaks are reached. However, we suggest that studying later life decline from the perspective of mutualism might prove a promising avenue for future work. If dynamic coupling is crucial for maintenance of cognitive abilities in later life, this may explain why declines are often strongly correlated (see Ghisletta & Lindenberger, 2003; Tucker-Drob, 2011, for further exploration of this hypothesis). Using large longitudinal cohorts and similar tests across the entire life span will allow for the investigation of possible “regime changes” within the same cohort.

Future work should study multiwave, multidomain cognitive data using principled model-selection methods to better capture the underlying dynamics of cognitive development. Data of high temporal resolution would allow one to move beyond group-level dynamics of individual differences to the ultimate goal, namely that of estimating individual differences in intraindividual dynamics over time. The investigation of individual coupling parameters across domains and across the life span is likely to yield a wealth of information on cognitive development in health and disease. The recent convergence of novel modeling techniques, large-scale data-gathering ability via tools such as smartphones, and the integration of behavioral data sets with data from neural and genetic sources of evidence together promise to provide new insight into some of the most elusive, yet fundamental, questions in cognitive psychology.

## Supplementary Material

Supplementary material

Supplementary material

Supplementary material

## Notes

1. BF_01_ refers to the default Bayes factor (Morey, Rouder, & Jamil, 2015) in favor of the null hypothesis.

2. These intercept parameters can be interpreted only in the context of the full LCS model that includes age as a covariate and the self-feedback pathway. The model-implied score increases in the absence of coupling were 0.370 (Vocabulary) and 0.559 (Matrix Reasoning); raw scores are shown in Table 1.
