# Supplementary material for: Mutualistic Coupling Between Vocabulary and Reasoning Supports Cognitive Development During Late Adolescence and Early Adulthood
Source: Psychol Sci. 2017 Aug 8;28(10):1419–31. doi: 10.1177/0956797617710785 (PMC5641983; doi:10.1177/0956797617710785)
Supplement: Supplementary material [file KievitSupplmentalMaterial_rev.doc]

**Acknowledgements**

RAK is supported by the Sir Henry Wellcome Trust (grant number 107392/Z/15/Z) and the UK Medical Research Council Programme (MC-A060-5PR61). The NSPN cohort was supported by a strategic award by the Wellcome Trust to the University of Cambridge and University College London (095844/Z/11/Z)

**Neuroscience in Psychiatry Network (NSPN) Consortium author list**

Chief investigator:

Ian Goodyer (Ed Bullmore from 01/01/2017)

Principal investigators:

Edward Bullmore (Ian Goodyer from 01/01/2017)

Raymond Dolan

Peter Fonagy

Peter Jones

Associated faculty:

Michael Moutoussis

Pasco Fearon

Anne-Laura van Harmelen

Petra Vértes

Kirstie Whitaker

Rogier Kievit

Project managers:

Becky Inkster

Gita Prabhu

Data managers:

Cinly Ooi

Barry Widmer

Research assistants:

Ayesha Alrumaithi

Sarah Birt

Kalia Cleridou

Hina Dadabhoy

Sian Granville

Elizabeth Harding

Alexandra Hopkins

Daniel Isaacs

Janchai King

Danae Kokorikou

Harriet Mills

Ciara O’Donnell

Sara Pantaleone

Administration team:

Junaid Bhatti

Laura Villis

**Conflicts of interest**

E.T.B. is employed half-time by the University of Cambridge and half-time by GlaxoSmithKline; he holds stock in GlaxoSmithKline. All authors report no additional perceived or actual conflicts of interest.
